# Circ-MBOAT2 knockdown represses tumor progression and glutamine catabolism by miR-433-3p/GOT1 axis in pancreatic cancer

**DOI:** 10.1186/s13046-021-01894-x

**Published:** 2021-04-08

**Authors:** Xiaoxiao Zhou, Kun Liu, Jing Cui, Jiongxin Xiong, Heshui Wu, Tao Peng, Yao Guo

**Affiliations:** Department of Pancreatic Surgery, Union Hospital, Tongji Medical College, Huazhong University of Science and Technology, No.1277 Jiefang Avenue, Wuhan, 430022 Hubei Province China

**Keywords:** Pancreatic cancer, Circ-MBOAT2, miR-433-3p, GOT1

## Abstract

**Background:**

Pancreatic cancer is a malignant tumor and ranks the sixth in incidence among cancers. Circular RNA (circRNA) has been reported to regulate the progression of pancreatic cancer. However, the effects of circ-membrane bound O-acyltransferase domain containing 2 (circ-MBOAT2) on regulating pancreatic cancer process were unclear.

**Methods:**

The expression levels of circ-MBOAT2, microRNA-433-3p (miR-433-3p) and glutamic-oxaloacetic transaminase 1 (GOT1) mRNA were detected by quantitative real-time polymerase chain reaction (qRT-PCR). GOT1 protein expression was determined by western blot analysis. Cell proliferation was illustrated by 3-(4,5)-dimethylthiahiazo (−z-y1)-3,5-di-phenytetrazoliumromide (MTT) and cell colony formation assay. Cell apoptosis was demonstrated by flow cytometry analysis. Cell invasion and migration were investigated by transwell invasion and wound-healing assays. Glutamine catabolism was explained by detecting glutamine consumption, alpha ketoglutarate (α-KG) production and glutamate production. In vivo assay was performed to illustrate the impacts of circ-MBOAT2 silencing on tumor formation in vivo. The binding relationship between miR-433-3p and circ-MBOAT2 or GOT1 was predicted by circinteractome or starbase online databases, and identified by dual-luciferase reporter assay.

**Results:**

Circ-MBOAT2 and GOT1 expression were significantly upregulated, while miR-433-3p expression was downregulated in pancreatic cancer tissues and cells compared with normal pancreatic tissues or cells. Circ-MBOAT2 silencing repressed cell proliferation, migration, invasion and glutamine catabolism, whereas promoted cell apoptosis in pancreatic cancer. Additionally, circ-MBOAT2 acted as a sponge of miR-433-3p, which was found to be associate with GOT1. MiR-433-3p inhibitors hindered circ-MBOAT2 silencing-mediated impacts on pancreatic cancer progression and glutamine catabolism. Furthermore, circ-MBOAT2 silencing repressed tumor formation in vivo.

**Conclusion:**

Circ-MBOAT2 modulated tumor development and glutamine catabolism by miR-433-3p/GOT1 axis in pancreatic cancer. This finding suggests that circ-MBOAT2 may be a therapeutic target for pancreatic cancer.

**Supplementary Information:**

The online version contains supplementary material available at 10.1186/s13046-021-01894-x.

## Background

Pancreatic cancer is a common malignant digestive system disease with poor prognosis and delayed diagnosis [1]. The incidence and death rate of pancreatic cancer cases are increasing worldwide, which causes a heavy burden to human health [2]. Although gemcitabine-based chemotherapy is the conventional therapeutic manner, the 5-year survival rate of pancreatic cancer sufferers is still low with only 6% [3]. As we known, early diagnosis and therapy are for reducing mortality, which can be achieved by seeking and developing new reliable targets.

Circular RNA (circRNA) is a novel RNA without protein coding capacity, featured by stability, conservatism and specificity in expression [4]. Researchers have revealed that circRNAs are highly expressed in the cytoplasm owing to their ability in absorbing microRNAs (miRNAs), which commonly function at the post-transcriptional level [5]. Disease-specific expression of circRNAs made them available as molecular markers for the treatment of cancers [6]. Additionally, circRNAs were enrolled in the evolution of diverse cancers [7], such as gastric carcinoma [8], breast cancer [9], lung carcinoma [10] and bladder cancer [11]. As previously reported, circ_0066147 contributed to cell growth and metastasis via upregulating p21-activated kinase 1 (PAK1) through acting as a sponge for miR-330-5p in pancreatic cancer [12]. Zhu and his colleagues indicated that circ_0006215 upregulation promoted the viability and migration of pancreatic cancer cells [13]. In this study, it was found that the expression of circ-membrane bound O-acyltransferase domain containing 2 (circ-MBOAT2) was significantly increased in pancreatic cancer specimen and cells; however, the underlying mechanism was still unclear.

MiRNAs are class of noncoding RNAs and are about 20 nucleotides in size [14]. MiRNAs act as oncogenes or anti-oncogenes in cancer progression by regulating cellular biological activities, such as cell proliferation, metastasis, apoptosis and glutamine catabolism [15, 16]. MiR-433-3p has been unveiled to repress tumor process. For example, miR-433-3p inhibited cell proliferation and metastasis, but contributed to cell apoptosis by binding to F-box protein 22 (FBXO22) in osteosarcoma [17] and through interacting with CREB in glioma [18]. MiR-433-3p inhibited cell epithelial-mesenchymal transition in colon cancer via targeting annexin A2 [19]. Bi et al. also found that miR-433 (miR-433-3p) sponged by LINC00657 restrained cell growth and facilitated cell apoptosis through restraining the expression of p21 (RAC1) activated kinase 4 in pancreatic ductal cancer [20]. In this experiment, it was found that miR-433-3p contained the binding sites of circ-MBOAT2. Whether circ-MBOAT2 could mediate pancreatic cancer growth via absorbing miR-433-3p was further explored in this study.

Glutamic-oxaloacetic transaminase 1 (GOT1) plays a vital role in the production of non-essential amino acids in glutamine metabolism, which is necessary for cell proliferation of various cancers [21, 22]. Research explained GOT1 served as a tumor promoter in pancreatic cancer growth [23]. Of note, starbase online database (http://starbase.sysu.edu.cn/agoClipRNA.php?source=mRNA) showed GOT1 contained the binding sequence of miR-433-3p. These data demonstrated GOT1 might be implicated in miR-433-3p-mediated pancreatic cancer progression. Thus, we further explored the relationship between miR-433-3p and GOT1 in modulating pancreatic cancer development.

In this paper, the impacts of circ-MBOAT2 silencing on cell proliferation, metastasis, apoptosis and glutamine metabolism were revealed. Whether circ-MBOAT2 was a sponge of miR-433-3p and miR-433-3p targeted GOT1 were disclosed. In addition, rescue experiments were employed to illustrate the effects between miR-433-3p and circ-MBOAT2 or GOT1 on tumor evolution and glutamine metabolism. Furthermore, the influences of circ-MBOAT2 knockdown on tumor formation in vivo were unveiled.

## Materials and methods

### Specimen collection and ethics committee

34 pairs of human pancreatic cancer and matched normal tissues were obtained from patients with pancreatic cancer from Union Hospital, Tongji Medical College, Huazhong University of Science and Technology. The collected pancreatic cancer tissues included stage I-II pancreatic cancer tissues (*N* = 22) and stage III-IV pancreatic cancer tissues (*N* = 12), and the clinical stage was confirmed by at least two pathologists. Collected tissues were stored at − 80 °C in a refrigerator. The Ethics Committee of Union Hospital, Tongji Medical College, Huazhong University of Science and Technology allowed this research. Relevant patients signed the written informed consents before surgery.

### Cell purchase and culture

EK-Bioscience Co., Ltd. (Guangzhou, China) provided human pancreatic cancer cell lines (AsPC-1, BxPC-3, PANC-1 and SW1990) and human pancreas ductal epithelial cell line HPDE. HPDE, AsPC-1 and BxPC-3 cells were grown in Roswell Park Memorial Institute-1640 (RPMI-1640; Procell, Wuhan, China). PANC-1 and SW1990 cells were cultivated in Dulbecco’s modified Eagle’s medium (DMEM; Procell) and L15 media (Procell), respectively. Media were supplemented with 10% fetal bovine serum (FBS; Procell) and 100 μg/mL penicillin-streptomycin solution (Procell). Cells were grown at 37 °C in a humid incubator with 5% CO_2_.

### Plasmid construction and cell transfection

Small interfering RNA targeting circ-MBOAT2 and GOT1 (si-circ-MBOAT2 and si-GOT1), miR-433-3p mimics and inhibitors (miR-433-3p and anti-miR-433-3p) and controls (si-NC, si-con, sh-NC, miR-NC and anti-miR-NC) were synthesized by GenePharma (Shanghai, China). The small hairpin RNA against circ-MBOAT2 was built by inserting top strand (5′-GATCCGAGAACATGCACAAGTCAACTCAAGAG AGTTGACTTGTGCATGTTCTCTTTTTG-3′) and bottom strand (5′-AATTCAAAAA GAGAACATGCACAAGTCAACTCACTTCAGTTGACTTGTGCATGTTCTCG-3′) into the pCDH-U6-MCS-EF1-GreenPuro vector, and named as sh-circ-MBOAT2. The overexpression plasmids of circ-MBOAT2 (circ-MBOAT2) and GOT1 (GOT1) and controls (pCD5-ciR and pcDNA) were provided by Geneseed (Guangzhou, China). Cell transfection was conducted with Lipofectamine 2000 (Thermo Fisher, Waltham, MA, USA). Si-circ-MBOAT2, si-GOT1, miR-433-3p, anti-miR-433-3p, circ-MBOAT2, GOT1 and their controls were used to determine that whether circ-MBOAT2 regulated pancreatic cancer development and glutamine catabolism by miR-433-3p/GOT1 axis. Sh-circ-MBOAT2 and sh-NC were employed to reveal circ-MBOAT2-mediated impacts on tumor formation in vivo. The synthesized oligonucleotide sequences were si-circ-MBOAT2 5′-GAGAACATGCACAAGTCAACT-3′, miR-433-3p 5′-AUCAUGAUGGGCUCCUCGGUGU-3′, anti-miR-433-3p 5′-ACACCGAGGAGCCCAUCAUGAU-3′, si-NC 5′-CCTCTACCTGTCGCTGAGCTGTAAT-3′, miR-NC 5′-UUUGUACUACACAAAAGUACUG-3′ and anti-miR-NC 5′-CAGUACUUUUGUGUAGUACAAA-3′.

### Quantitative real-time polymerase chain reaction (qRT-PCR)

Collected tissues or cultured cells were isolated using miRNeasy Mini Kit (Qiagen, Valencia, CA, USA). The concentration of obtained RNAs was detected with NanoDrop-1000 apparatus (Thermo Fisher). Then, cDNA was synthesized with a High-Capacity cDNA RT Kit (Thermo Fisher) or MicroRNA RT Kit (Thermo Fisher). For determining relative levels of RNAs/mRNAs, a SuperReal PreMix Color kit (Tiangen, Beijing, China) was employed with a Mx3000P system (Stratagene, Santa Clara, CA, USA). Obtained data were analyzed with the 2^-∆∆Ct^ method, and U6 and β-actin acted as references. The sense and antisense primers were circ-MBOAT2 5′-ATGCCTTACACTTTCTTG-3′ and 5′-GAGCTAGTTTTGCTTGAA-3′; linear MBOAT2 5′-GATGTTTCGGAAGGATGA-3′ and 5′-TTGTAAGAGCAAAGTGGG-3′; miR-433-3p 5′-ACACTCCAGCTGGGATCATGATGGGCTCCT-3′ and 5′-TGGTGTCGTGGAGTCG-3′; miR-144-3p 5′-ACACTCCAGCTGGGTACAGTATAGATGA-3′ and 5′-TGGTGTCGTGGAGTCG-3′; GOT1 5′-CTGGGAGTGGGAGCATAT-3′ and 5′-CAAGGGCAAGACGAGAAG-3′; U6 5′-CTCGCTTCGGCAGCACA-3′ and 5′-AACGCTTCACGAATTTGCGT-3′; glyceraldehyde 3-phosphate dehydrogenase (GAPDH) 5′-GGTCACCAGGGCTGCTTT-3′ and 5′-GGAAGATGGTGATGGGATT-3′; β-actin 5′-CACCATTGGCAATGAGCGGTTC-3′ and 5′-AGGTCTTTGCGGATGTCCACGT-3′.

### RNase R treatment assay

Cultured PANC-1 and SW1990 cells were collected and lysed. RNAs were extracted and then incubated with RNase R (3 U/μg RNA, Geneseed) at 37 °C for 30 min. After that, RNAs were purified using RNeasy MinElute Cleaning Kit (Qiagen). Finally, circ-MBOAT2 expression was determined by qRT-PCR. Linear MBOAT2 functioned as a control.

### Cytoplasmic and nuclear circ-MBOAT2 analysis

Cytoplasmic and nuclear circ-MBOAT2 were assessed with a PARIS™ Kit (Thermo Fisher). In short, fresh PANC-1 and SW1990 cells were harvested and suspended in 200 μL ice-cold Cell Fractionation Buffer (Thermo Fisher), which were then centrifuged at 450 rpm for 4 min. The cytoplasmic fraction and nuclear pellet were carefully separated and lysed. Finally, RNAs were isolated, and circ-MBOAT2 expression was determined by qRT-PCR. U6 and GAPDH served as references.

### 3-(4,5)-dimethylthiahiazo (−z-y1)-3,5-di-phenytetrazoliumromide (MTT) assay

Cell viability was illustrated by a MTT kit (Solarbio, Beijing, China). In short, after performing various treatments, PANC-1 and SW1990 cells were cultured in 96-well plates for 24, 48 and 72 h, respectively. Fresh media (Procell) were mixed with MTT solution (Solarbio) and severally added into culture plates. 4 h later, dimethyl sulfoxide (Sigma, St. Louis, MO, USA) was added into wells to dissolve formazan. Samples were assessed by microplate reader (Thermo Fisher), and results were revealed by analyzing the output of the wavelength at 490 nm.

### Cell colony formation assay

PANC-1 and SW1990 cells were seeded in 6-well plates. Media were renewed every 3 days during culture. Two weeks later, cell supernatant was removed, and proliferative colonies were incubated with paraformaldehyde (Sigma) and crystal violet (Sigma), respectively. Cell colony-forming ability was illustrated via counting cell numbers. A colony was deemed when cell numbers > 50.

### Flow cytometry analysis

Cell apoptosis was determined by an Annexin V-fluorescein isothiocyanate (Annexin V-FITC) detection kit (Solarbio). Briefly, cultured cells were digested with trypsin (Thermo Fisher), and suspended in binding buffer (Solarbio). Following that, Annexin V-FITC (Solarbio) and propidium iodide (PI; Solarbio) were severally incubated with cells in dark. Finally, samples were analyzed with flow cytometer (Thermo Fisher).

### Transwell invasion assay

The invasion of PANC-1 and SW1990 cells was investigated with transwell chambers with Matrigel (Corning, Madison, New York, USA). Shortly, cells were seeded in the upper chambers with FBS-free DMEM or L15 media (Procell). And DMEM or L15 media containing 15% FBS (Procell) were added into lower chambers. At 24 h after culture, cell supernatant was discarded and cells were singly incubated with methanol (Sigma) as well as crystal violet (Sigma). Results were analyzed by figuring up the cell numbers in the lower chambers under microscope (Nikon, Tokyo, Japan) at a 100(×) magnification.

### Wound-healing assay

PANC-1 and SW1990 cells were cultured in 6-well plates until their confluence reached about 100%. Then, cell wounds were created and cells were washed using phosphate buffer solution (PBS; Thermo Fisher). Cells were continued to be cultured in serum-free media for 24 h. Finally, cell migratory ability was determined via calculating the width of wounds under microscope (Nikon) with a 100(×) magnification.

### The determination of glutamine uptake and α-KG production

Glutamine uptake and α-KG production were determined by glutamine and α-KG assay kits (Abcam, Cambridge, UK), respectively. In brief, cultured cells were harvested and then suspended in assay Buffer (Abcam). After that, samples were centrifuged at 9000 rpm for 12 min, and supernatant was collected. Perchloric acid (Abcam) and potassium hydroxide (Abcam) were used to incubate with supernatant, respectively. After performing centrifugation at 12,000 rpm for 12 min, supernatant was collected and analyzed with the microplate reader (Thermo Fisher) with the wavelength at 450 nm for glutamine uptake assay or at 570 nm for α-KG production assay.

### The detection of glutamate production

Glutamate production was detected with glutamate assay kit (Abcam). Briefly, cells were collected when their confluence reached about 80%. Then, harvested cells were incubated with lysis buffer (Abcam) for 15 min. After that, Assay buffer (Abcam), Enzyme mixture (Abcam) and NADP Stock Solution (Abcam) were mixed with lysates. Finally, samples were analyzed with the microplate reader (Thermo Fisher) with the wavelength at 570 nm.

### In vivo assay

Charles River (Beijing, China) provided 5-week old nude mice (*N* = 16), and mice were fed in pathogen-free environment. Nude mice were averagely divided into 2 groups. 4 × 10^6^ SW1990 cells stably transfected with sh-circ-MBOAT2 or sh-NC were diluted in 0.2 mL PBS, and then injected into the right flank of the back of mice. Eight days later, tumor volume was measured every 3 days. Twenty-three days later, nude mice were killed and tumor weight was determined. Additionally, a part of every tumor was reserved for further analyzing in circ-MBOAT2 expression. The Animal Care Committee of Union Hospital, Tongji Medical College, Huazhong University of Science and Technology approved this study.

### Dual-luciferase reporter assay

According to the results predicted by circinteractome online database (https://circinteractome.nia.nih.gov/index.html) or starbase online database (http://starbase.sysu.edu.cn/agoClipRNA.php?source=mRNA), the wild-type (WT) plasmids and mutant vectors of circ-MBOAT2 and GOT1 3′-untranslated region (3’UTR) were provided by Geneseed Co., Ltd., and severally named as WT-circ-MBOAT2, GOT1 3’UTR-WT, MUT-circ-MBOAT2 and GOT1 3’UTR-MUT. Constructed plasmids were transfected into PANC-1 and SW1990 cells with miR-433-3p or miR-NC after mixed with DharmaFECT 4 (Thermo Fisher) according to the manufacturer’s instructions. Luciferase activities were detected by Dual-Lucy Assay Kit (Solarbio) with *Renilla* luciferase activity as a control.

### Western blot analysis

Collected tissues and cultured cells were severally lysed with RIPA buffer (Beyotime, Shanghai, China). Then, lysates were mixed with loading buffer (Thermo Fisher), and were then boiled in boiling water for 8 min. Lysates were loaded on 12% bis-tris-acrylamide gel (Thermo Fisher) to separate protein. After that, protein bands were electrotransferred onto polyvinylidene fluoride membranes (Millipore, Bradford, MA, USA), which were then immersed in nonfat milk (Solarbio). Membranes were incubated with anti-GOT1 (1:1000; Affinity, Nanjing, China) and anti-β-actin (1:8000; Affinity) overnight at 4 °C, and were then incubated with horseradish peroxidase-marked secondary antibody (1:8000; Affinity) at 37 °C for 2 h. Protein bands were presented by eyoECL Plus Kit (Beyotime). β-actin acted as a reference.

### Statistical analysis

Data from 3 independent duplicate tests were assessed with SPSS 21.0 software (IBM, Somers, NY, USA), and were presented as means ± standard deviations (SD). The linear relationship between miR-433-3p and circ-MBOAT2 or GOT1 was compared with Spearman’s correlation test. Two-tailed Student’s *t*-tests or Wilcoxon rank-sum test was employed to assess the significant differences between the two groups, and one-way analysis of variance (ANOVA) was performed to compare the difference among 3 groups or more groups. Statistical significance was deemed when *P* value < 0.05.

## Results

### Circ-MBOAT2 expression was upregulated in the tissues and cells of pancreatic cancer

**Fig. 1:**
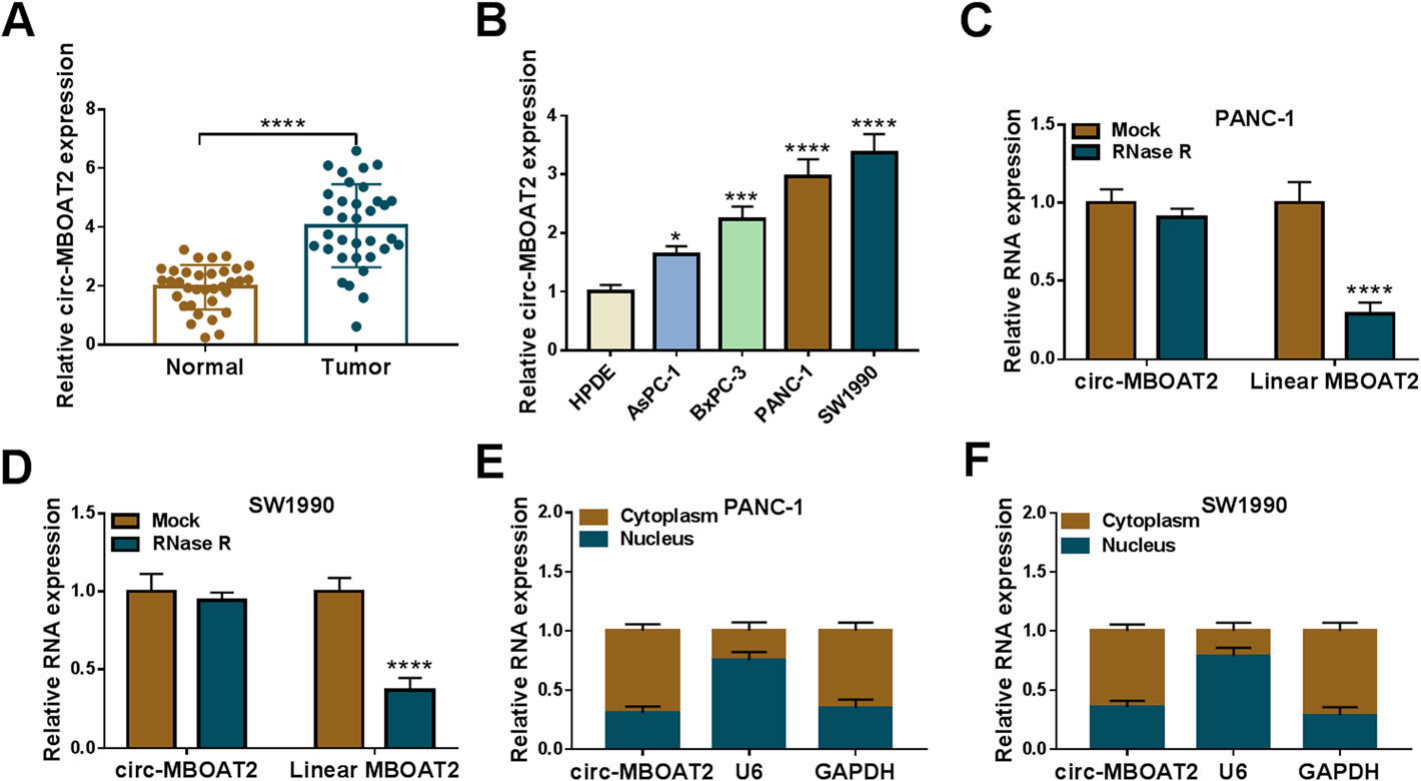
Circ-MBOAT2 was overexpressed in the tissues and cells of pancreatic cancer. **a** and **b** The expression level of circ-MBOAT2 was determined by qRT-PCR in 34 pairs of pancreatic cancer and paracancerous normal pancreatic tissues as well as HPDE, AsPC-1, BxPC-3, PANC-1 and SW1990 cells. **c** and **d** RNase R treatment assay was employed to illustrate the stability of circ-MBOAT2. **e** and **f** Cytoplasmic and nuclear circ-MBOAT2 analysis assay was performed to demonstrate that circ-MBOAT2 was mainly located in cytoplasm. The β-actin was employed for the normalization of circ-MBOAT2/MBOAT2/GAPDH, and U6 was used for U6. *P < 0.05, ***P < 0.001 and ****P < 0.0001

Circ-MBOAT2 expression was firstly determined in the tissues and cells of pancreatic cancer. Results showed that circ-MBOAT2 expression was dramatically increased in tumors and AsPC-1, BxPC-3, PANC-1 and SW1990 cells when compared with normal tissues and HPDE cells, respectively (Fig. 1a and b). PANC-1 and SW1990 cells were chosen in further experiments as the dramatically highest expression of circ-MBOAT2 in them. Additionally, qRT-PCR data displayed that circ-MBOAT2 expression was higher in stage III-IV pancreatic cancer tissues than in stage I-II (Figure S1). Subsequently, RNase R treatment assay presented that circ-MBOAT2 expression had no apparent change after RNase R treatment, whereas the expression of linear MBOAT2 was significantly downregulated (Fig. 1c and d), implicating circ-MBOAT2 was more stable than linear MBOAT2. Furthermore, data presented that circ-MBOAT2 expression was higher in cytoplasm than in nucleus (Fig. 1e and f), which suggested that circ-MBOAT2 was mainly located in cytoplasm. These results illustrated circ-MBOAT2 might be enrolled in the progression of pancreatic cancer.

### Circ-MBOAT2 silencing repressed cell proliferation, migration, invasion and glutamine metabolism, while induced cell apoptosis in pancreatic cancer in vitro

**Fig. 2:**
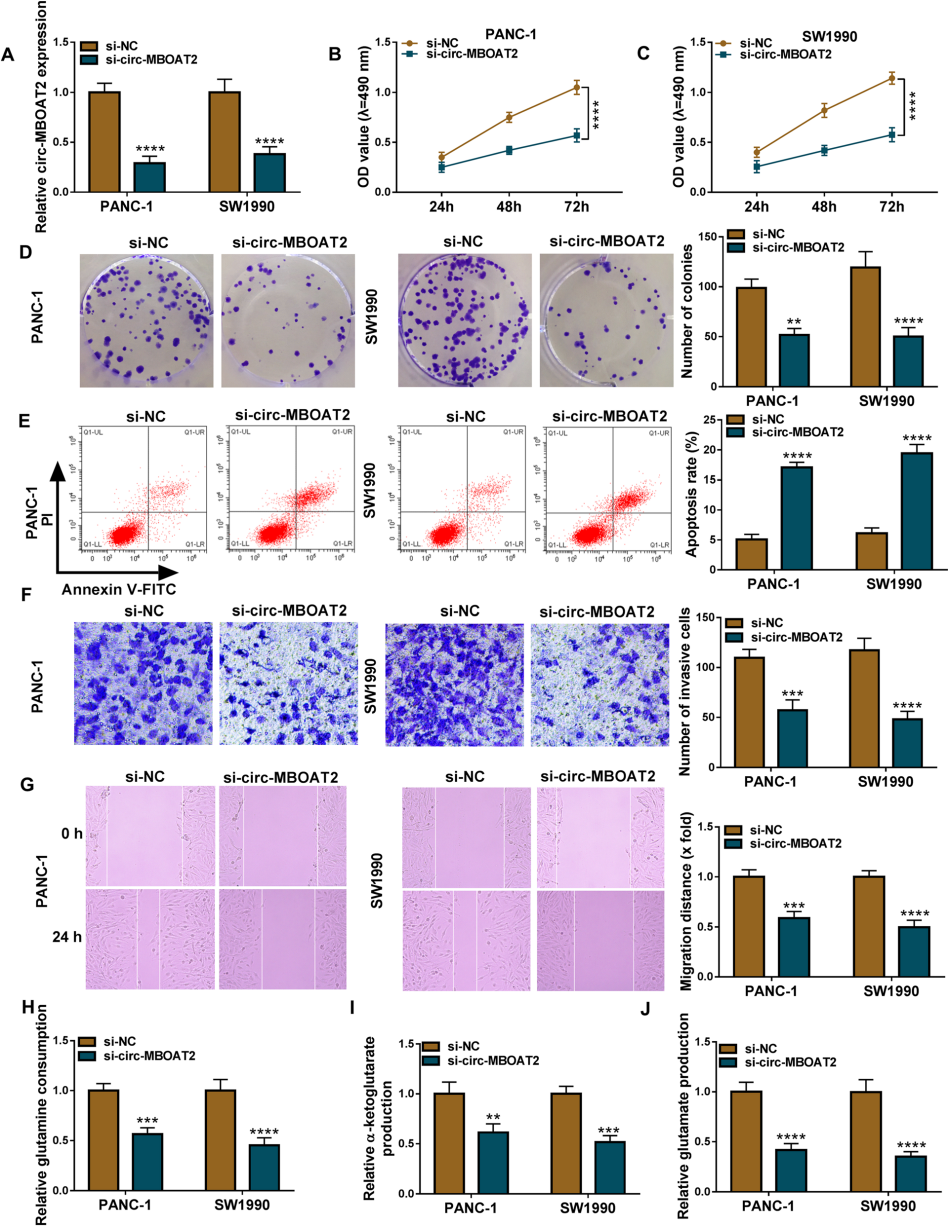
Circ-MBOAT2 absence restrained the process of pancreatic cancer. **a** The impact of circ-MBOAT2 knockdown on the expression of circ-MBOAT2 was determined by qRT-PCR in PANC-1 and SW1990 cells with β-actin as an internal reference. **b**-**d** MTT and cell colony formation assays were employed to reveal the impact of circ-MBOAT2 silencing on the proliferation of PANC-1 and SW1990 cells. **e** Flow cytometry analysis was performed to illustrate the impact of circ-MBOAT2 knockdown on the apoptosis of PANC-1 and SW1990 cells. **f** and **g** The impacts of circ-MBOAT2 downregulation on the invasion and migration of PANC-1 and SW1990 cells were demonstrated by transwell invasion and wound-healing assays, respectively. **h**-**j** Glutamine, α-KG and glutamate assay kits were used to detect glutamine consumption, α-KG production and glutamate production, respectively, in PANC-1 and SW1990 cells transfected with si-circ-MBOAT2 or si-NC. **P < 0.01, ***P < 0.001 and ****P < 0.0001

The study continued to explore that whether circ-MBOAT2 regulated the development of pancreatic cancer. Results firstly showed circ-MBOAT2 expression was notably downregulated in PANC-1 and SW1990 cells transfected with si-circ-MBOAT2 compared with the cells transfected with si-NC (Fig. 2a). Meanwhile, there was no change in MBOAT2 expression in si-circ-MBOAT2-transfected PANC-1 and SW1990 cells in comparison with the two types of cells transfected with si-NC (Figure S2). The above data implicated si-circ-MBOAT2 was effective in downregulating circ-MBOAT2 expression. Subsequently, MTT and cell colony formation assays presented that circ-MBOAT2 silencing inhibited cell viability and colony-forming ability (Fig. 2b-d), which meant that circ-MBOAT2 absence hindered cell proliferation in PANC-1 and SW1990 cells. Flow cytometry analysis also explained circ-MBOAT2 knockdown induced the apoptosis of PANC-1 and SW1990 cells (Fig. 2e). The invasion and migration of PANC-1 and SW1990 cells were also suppressed after circ-MBOAT2 silencing in PANC-1 and SW1990 cells (Fig. 2f and g). The impact of circ-MBOAT2 knockdown on glutamine metabolism was further revealed. Results displayed circ-MBOAT2 downregulation inhibited the consumption of glutamine and the production of α-KG and glutamate (Fig. 2h-j), suggesting circ-MBOAT2 silencing repressed glutamine metabolism.

Furthermore, rescue experiments were performed to determine the effects of circ-MBOAT2 overexpression on si-circ-MBOAT2-mediated pancreatic cancer cell processes. And results showed that circ-MBOAT2 overexpression attenuated si-circ-MBOAT2-mediated repression on circ-MBOAT2 expression (Figure S3A). The inhibitory effects of circ-MBOAT2 silencing on the cell viability and colony formation ability were also impaired after circ-MBOAT2 overexpression (Figure S3B-D). Similarly, si-circ-MBOAT2-triggered influences on cell apoptosis, invasion, migration, glutamine, α-KG and glutamate were also restrained by ectopic circ-MBOAT2 expression (Figure S3E-J). These data investigated circ-MBOAT2 knockdown inhibited tumor development and glutamine metabolism of pancreatic cancer.

### Circ-MBOAT2 knockdown repressed tumor growth in vivo

**Fig. 3:**
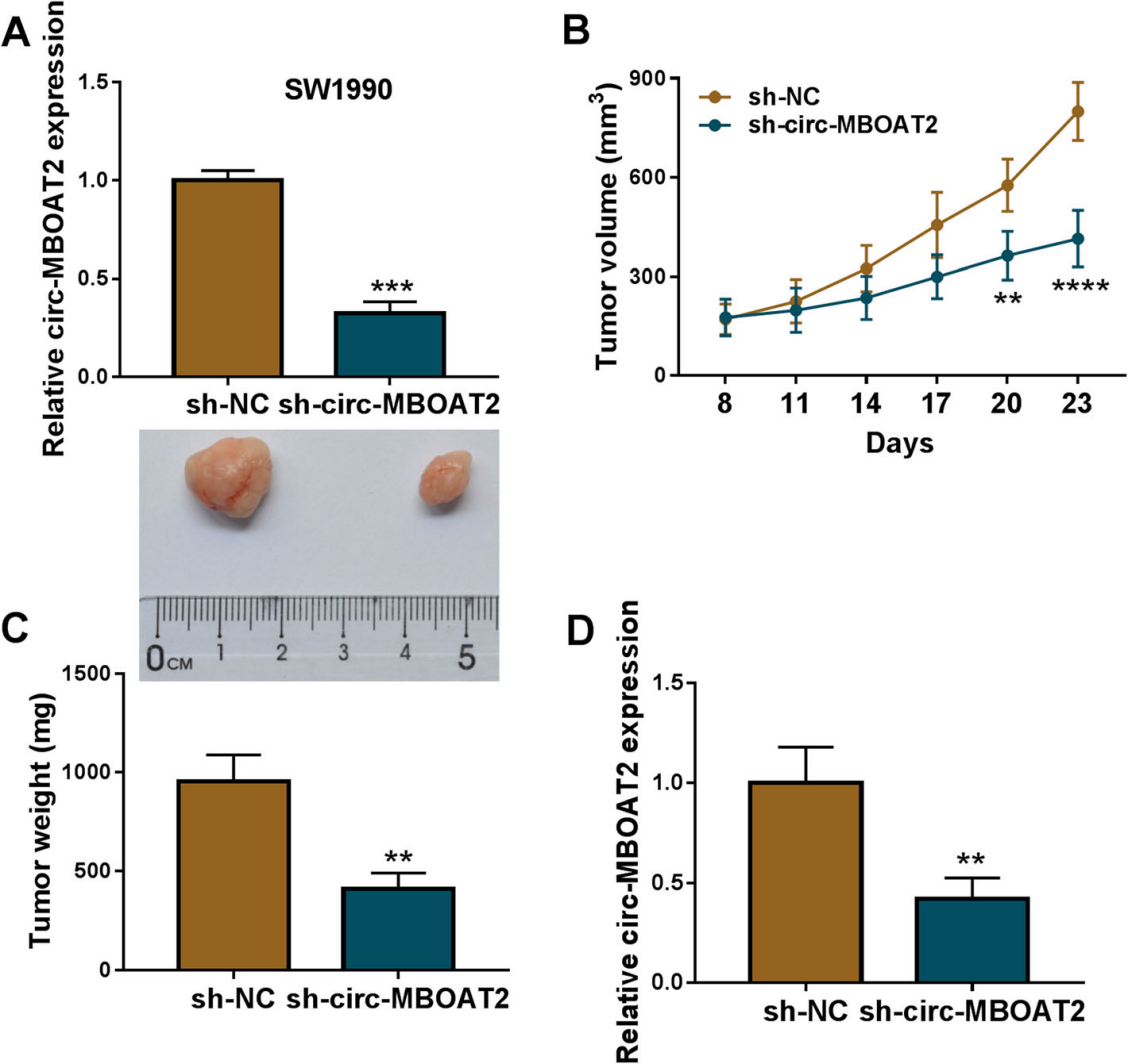
Circ-MBOAT2 regulated tumor growth in vivo. **a** Circ-MBOAT2 expression was determined by qRT-PCR in SW1990 cell line transfected with sh-circ-MBOAT2 or sh-NC with β-actin as an internal reference. **b** and **c** The impacts of circ-MBOAT2 silencing on the volume and weight of tumors were unveiled. **d** The effect of circ-MBOAT2 silencing on circ-MBOAT2 expression was revealed by qRT-PCR in vivo with β-actin as an internal reference. **P < 0.01, ***P < 0.001 and ****P < 0.0001

The impacts of circ-MBOAT2 silencing on tumor formation in vivo were further disclosed. In order to illustrate that, the SW1990 cell line stably transfected with sh-circ-MBOAT2 or sh-NC was severally established. And qRT-PCR results showed circ-MBOAT2 expression was significantly downregulated in SW1990 cells transfected with sh-circ-MBOAT2 compared with the cells transfected with sh-NC (Fig. 3a), suggesting the SW1990 cell line stably transfected with sh-circ-MBOAT2 was successfully established. Subsequently, the volume and weight of the forming tumors were smaller or lighter in sh-circ-MBOAT2 group than in sh-NC group (Fig. 3b and c), showing the repressive impact of circ-MBOAT2 silencing on tumor formation. Furthermore, circ-MBOAT2 expression was dramatically decreased in the neoplasms from sh-circ-MBOAT2 group when compared with the tumors from sh-NC group (Fig. 3d), illustrating sh-circ-MBOAT2 was effective in downregulating MBOAT2 expression in the forming tumors. These data demonstrated circ-MBOAT2 absence inhibited tumor growth in vivo.

### Circ-MBOAT2 acted as a sponge of miR-433-3p

**Fig. 4:**
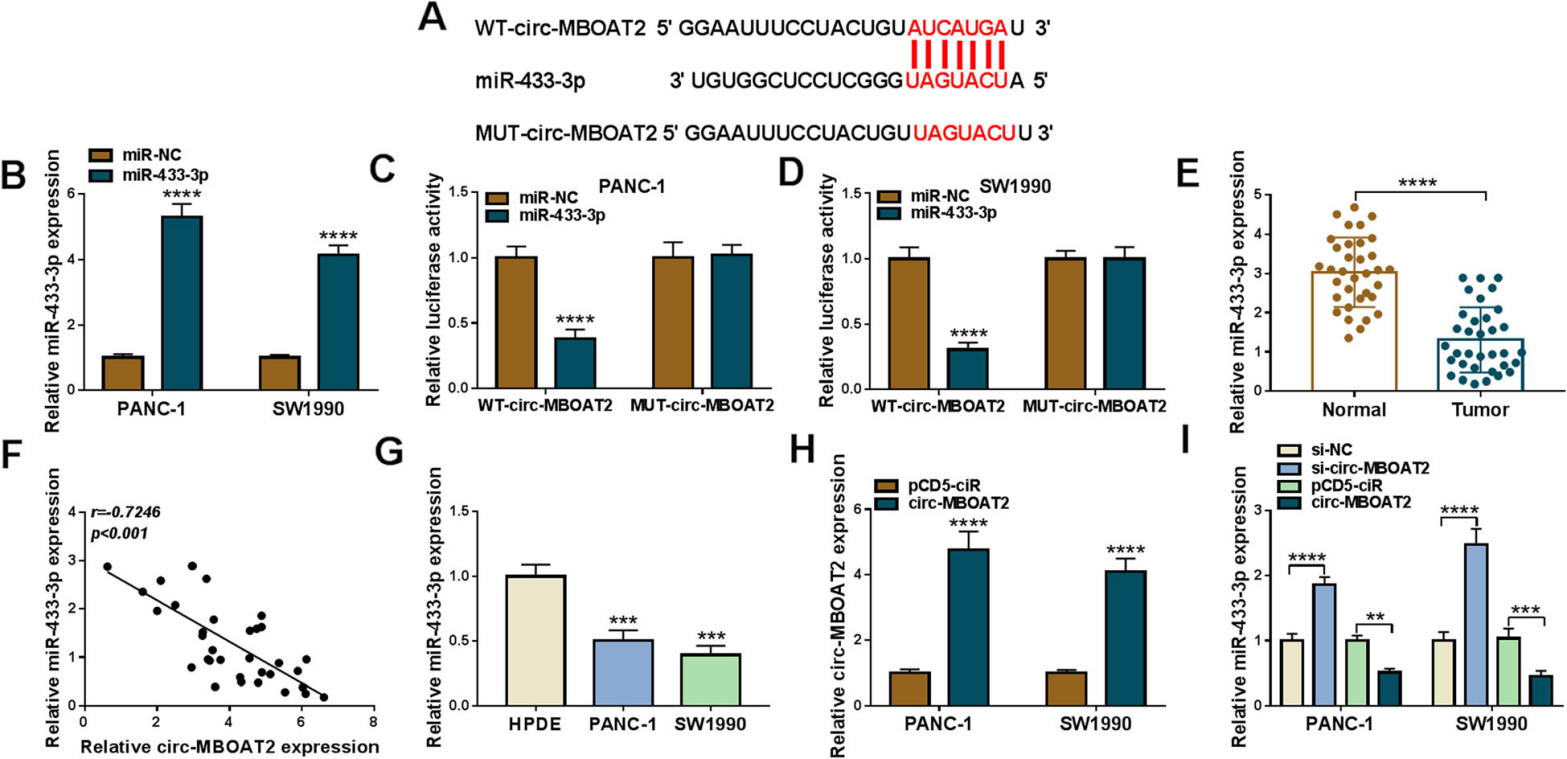
Circ-MBOAT2 was associated with miR-433-3p in pancreatic cancer cells. **a** The supposed binding sites between circ-MBOAT2 and miR-433-3p were predicted by circinteractome online database. **b** The overexpression efficiency of miR-433-3p was determined by qRT-PCR in PANC-1 and SW1990 cells with U6 as an internal reference. **c** and **d** The associated relationship between circ-MBOAT2 and miR-433-3p was illustrated by dual-luciferase reporter assay. **e** and **g** MiR-433-3p expression was detected by qRT-PCR in 34 pairs of pancreatic cancer and adjacent normal pancreatic tissues as well as HPDE, PANC-1 and SW1990 cells with U6 as an internal reference. **f** Spearman correlation analysis was employed to assess the linear relationship between circ-MBOAT2 expression and miR-433-3p expression with β-actin/U6 as internal references. **h** The overexpression efficiency of circ-MBOAT2 was determined by qRT-PCR in PANC-1 and SW1990 cells with β-actin as an internal reference. **i** The impacts of circ-MBOAT2 silencing and overexpression on miR-433-3p expression were determined by qRT-PCR in PANC-1 and SW1990 cells. **P < 0.01, ***P < 0.001 and ****P < 0.0001

The underneath mechanism by which circ-MBOAT2 regulated pancreatic cancer process was continued to be unveiled. Based on the expression property and biological function of circ-MBOAT2 in pancreatic cancer, circ-MBOAT2-associated miRNAs should exhibit low expression and anti-cancer role in pancreatic cancer. Thus, we screened all miRNAs containing the binding sites of circ-MBOAT2, and found miR-144-3p and miR-433-3p were suitable; however, further data showed circ-MBOAT2 silencing more upregulated miR-433-3p compared with the results in miR-144-3p expression (Figure S4). Thus, miR-433-3p was employed in subsequent study. Circinteractome online database showed the binding sequence between miR-433-3p and circ-MBOAT2 (Fig. 4a). In order to illustrate the supposed associated relationship between circ-MBOAT2 and miR-433-3p, the overexpression efficiency of synthesized miR-433-3p mimics was firstly detected. QRT-PCR data presented miR-433-3p expression was dramatically upregulated after miR-433-3p mimics transfection in PANC-1 and SW1990 cells (Fig. 4b), implicating miR-433-3p mimics was effective in upregulating miR-433-3p expression. Subsequently, dual-luciferase reporter assay showed the relative luciferase activity was dramatically repressed in WT-circ-MBOAT2 + miR-433-3p group, but there was no apparent change in MUT-circ-MBOAT2 + miR-433-3p group (Fig. 4c and d). It was found that miR-433-3p expression was significantly downregulated in pancreatic cancer tissues and PANC-1 and SW1990 cells as compared to normal tissues and HPDE cells, respectively (Fig. 4e and g). In addition, Spearman correlation analysis exhibited miR-433-3p expression was negatively related to circ-MBOAT2 expression (Fig. 4f). The effects of circ-MBOAT2 silencing and overexpression on miR-433-3p expression were further disclosed. QRT-PCR firstly showed the overexpression plasmid of circ-MBOAT2 was successfully built because of obviously high expression of circ-MBOAT2 after circ-MBOAT2 transfection (Fig. 4h). And we found miR-433-3p expression was apparently upregulated by si-circ-MBOAT2 and was significantly downregulated by circ-MBOAT2 (Fig. 4i). The above results investigated circ-MBOAT2 acted as a sponge of miR-433-3p.

### Circ-MBOAT2 regulated the progression of pancreatic cancer and glutamine metabolism by absorbing miR-433-3p

**Fig. 5:**
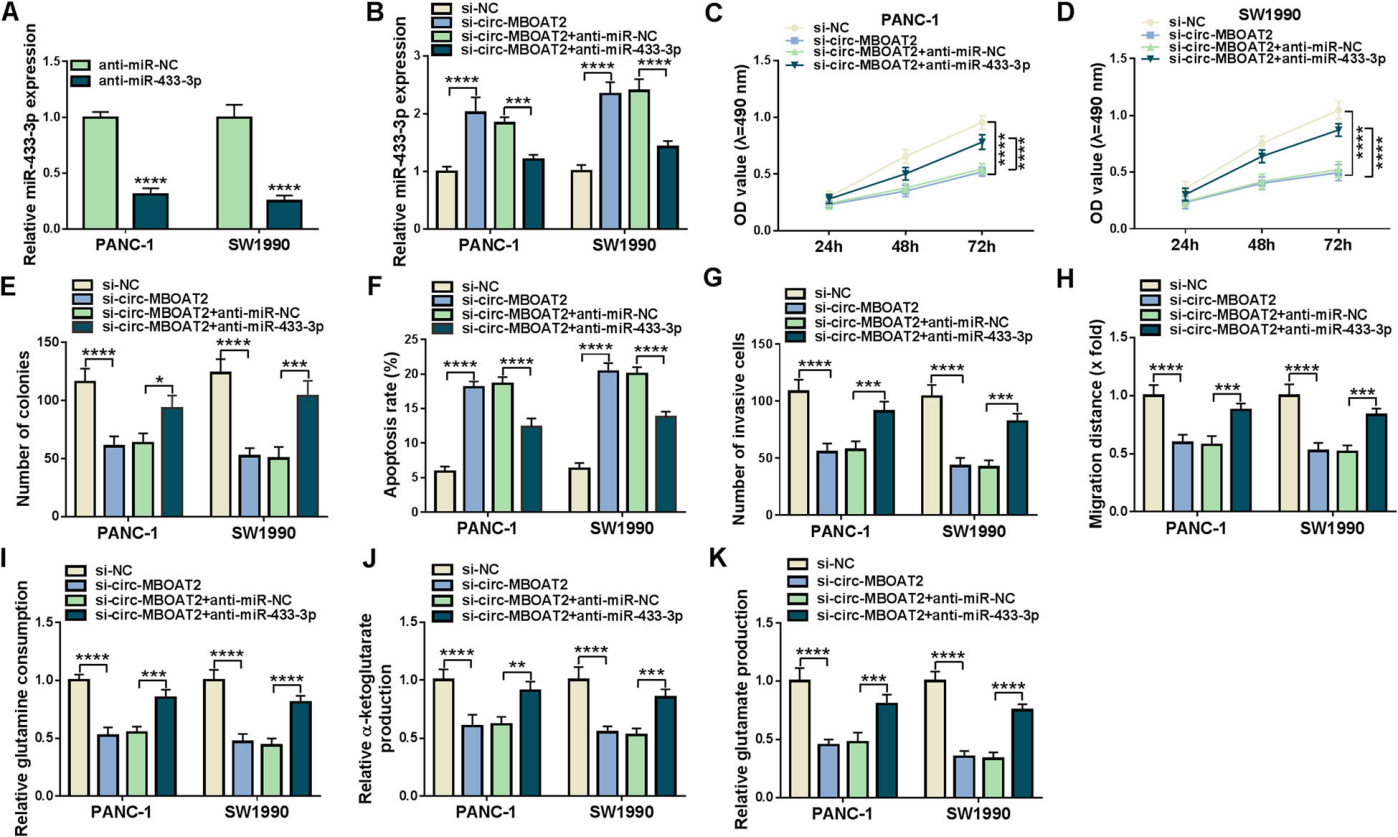
Circ-MBOAT2 downregulation repressed pancreatic cancer progression by binding to miR-433-3p. **a** The effect of miR-433-3p inhibitors on miR-433-3p expression was presented by qRT-PCR in PANC-1 and SW1990 cells. **b** The impacts between circ-MBOAT2 silencing and miR-433-3p inhibitors on miR-433-3p expression were illustrated by qRT-PCR. **c** and **d** The impacts between circ-MBOAT2 repression and miR-433-3p inhibitors on cell viability were revealed by MTT assay. **e** The impacts between circ-MBOAT2 repression and miR-433-3p inhibitors on cell colony-forming ability were revealed by cell colony formation assay. **f** Flow cytometry analysis was employed to explain the effects between circ-MBOAT2 silencing and miR-433-3p inhibitors on the apoptosis of PANC-1 and SW1990 cells. **g** and **h** The influences between circ-MBOAT2 repression and miR-433-3p inhibitors on the invasion and migration of PANC-1 and SW1990 cells were disclosed by transwell invasion and wound-healing assays, respectively. **i** Glutamine assay kit was utilized to illustrate the effects between circ-MBOAT2 silencing and miR-433-3p inhibitors on glutamine consumption in PANC-1 and SW1990 cells. **j** α-KG assay kit was used to present the effects between circ-MBOAT2 silencing and miR-433-3p inhibitors on α-KG production. **k** Glutamate assay kit was utilized to illustrate the effects between circ-MBOAT2 silencing and miR-433-3p inhibitors on glutamate production in PANC-1 and SW1990 cells. The U6 and β-actin were employed for the normalization of miR-433-3p and circ-MBOAT2, respectively. *P < 0.05, **P < 0.01, ***P < 0.001 and ****P < 0.0001

Whether circ-MBOAT2 modulated pancreatic cancer process via sponging miR-433-3p was further revealed. QRT-PCR results firstly showed miR-433-3p expression was dramatically decreased after anti-miR-433-3p transfection (Fig. 5a), which meant that anti-miR-433-3p was effective in reducing miR-433-3p expression. Subsequently, miR-433-3p was found its expression was notably upregulated after circ-MBOAT2 silencing, whereas this effect was restored by anti-miR-433-3p (Fig. 5b). MiR-433-3p inhibitors attenuated the repressive impacts of circ-MBOAT2 knockdown on the viability and colony-forming ability of PANC-1 and SW1990 cells (Fig. 5c-e). The promotion influence of circ-MBOAT2 knockdown on cell apoptosis was also restored after transfection of miR-433-3p inhibitors (Fig. 5f). In addition, miR-433-3p inhibitors impaired the inhibitory effects of circ-MBOAT2 silencing on the invasion and migration of PANC-1 and SW1990 cells (Fig. 5g and h). The repressive impacts of circ-MBOAT2 silencing on glutamine consumption, α-KG production and glutamate production were also reversed by miR-433-3p inhibitors (Fig. 5i-k). Thus, these evidences illustrated circ-MBOAT2 could regulate pancreatic cancer development and glutamine metabolism by absorbing miR-433-3p.

### MiR-433-3p was associated with GOT1 in pancreatic cancer cells

**Fig. 6:**
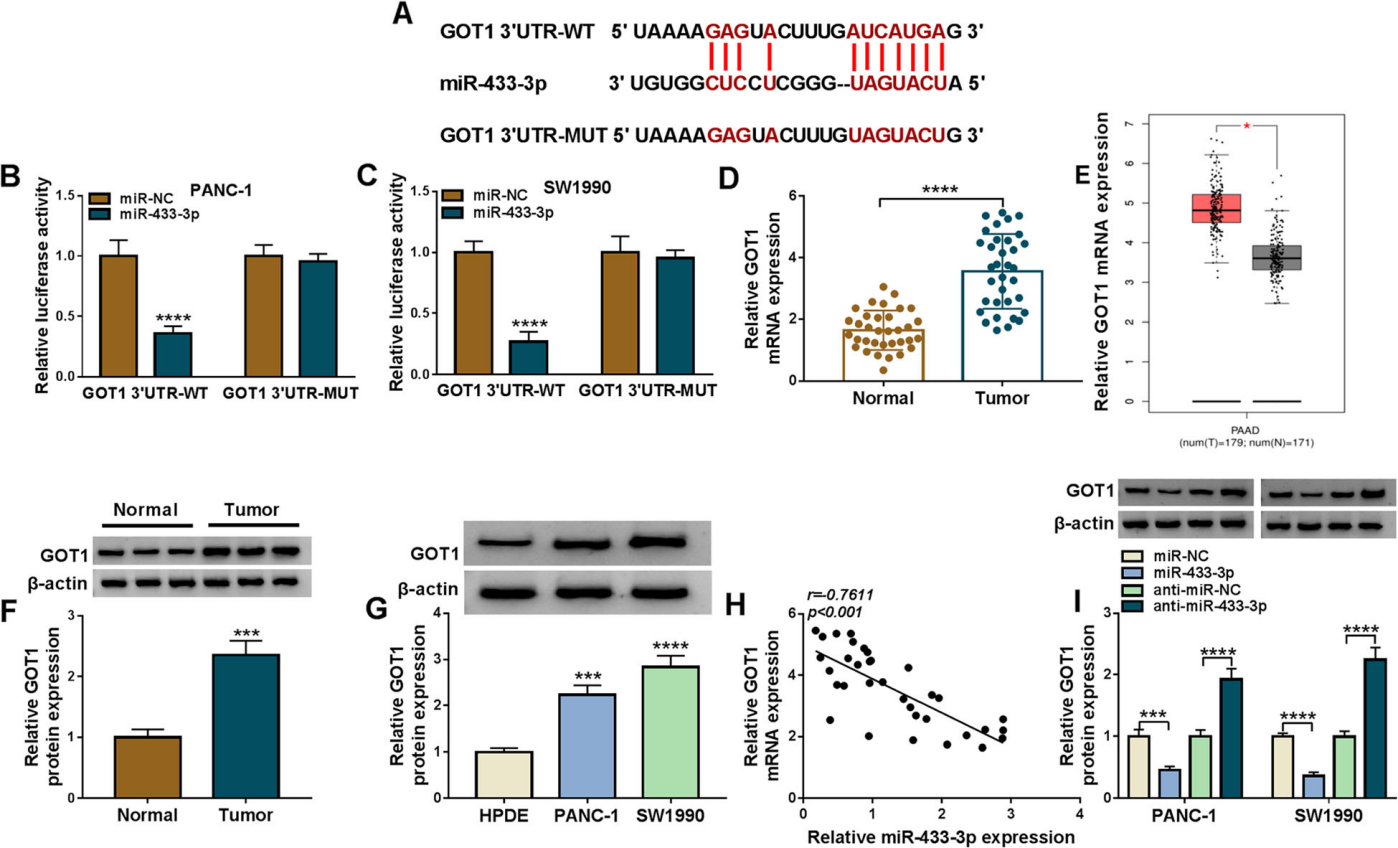
MiR-433-3p targeted GOT1 in PANC-1 and SW1990 cells. **a** The putative binding sites between miR-433-3p and GOT1 were predicted by starbase online database. **b** and **c** Dual-luciferase reporter assay was carried out to demonstrate the associated relationship between miR-433-3p and GOT1. **d** QRT-PCR was performed to detect GOT1 mRNA expression in 34 pairs of pancreatic cancer and adjacent normal pancreatic tissues. **e** UALCAN dataset was used to predict GOT1 expression in pancreatic cancer tissues (*N* = 179) and paracancerous normal tissues (*N* = 171). **f** and **g** Western blot analysis was performed to detect GOT1 protein expression in 23 pairs of pancreatic cancer and matched normal tissues as well as HPDE, PANC-1 and SW1990 cells. **h** Spearman correlation analysis was carried out to illustrate the linear relationship between GOT1 expression and miR-433-3p expression. **i** The impacts of miR-433-3p mimics and inhibitors on GOT1 protein expression were determined by western blot analysis. The U6 and β-actin were employed for the normalization of miR-433-3p and GOT1, respectively. *P < 0.05, ***P < 0.001 and ****P < 0.0001

The mechanism underlying miR-433-3p regulating pancreatic cancer development was revealed in this part. Starbase online database presented miR-433-3p possessed the binding sites of GOT1 (Fig. 6a). Meanwhile, dual-luciferase reporter assay showed the relative luciferase activity of miR-433-3p + GOT1 3’UTR-WT was obviously repressed, while that of miR-433-3p + GOT1 3’UTR-MUT group had no apparent change (Fig. 6b and c), suggesting that miR-433-3p could interact with GOT1 by binding to GOT1 3’UTR. Subsequently, data also displayed the mRNA and protein expression of GOT1 were greatly increased in pancreatic cancer tissues as well as PANC-1 and SW1990 cells compared with paracancerous normal tissues and HPDE cells, respectively (Fig. 6d, f and g). The significantly high expression of GOT1 in pancreatic cancer tissues also was found in UALCAN dataset (Fig. 6e). GOT1 was revealed its expression was negatively correlated with miR-433-3p expression (Fig. 6h). Additionally, GOT1 protein expression was obviously downregulated after transfection of miR-433-3p mimics and was notably upregulated by miR-433-3p inhibitors (Fig. 6i), showing that miR-433-3p inversely regulated GOT1 at the protein level. These results demonstrated miR-433-3p targeted GOT1.

### MiR-433-3p inhibited cell proliferation, migration, invasion and glutamine metabolism, whereas promoted cell apoptosis by targeting GOT1

**Fig. 7:**
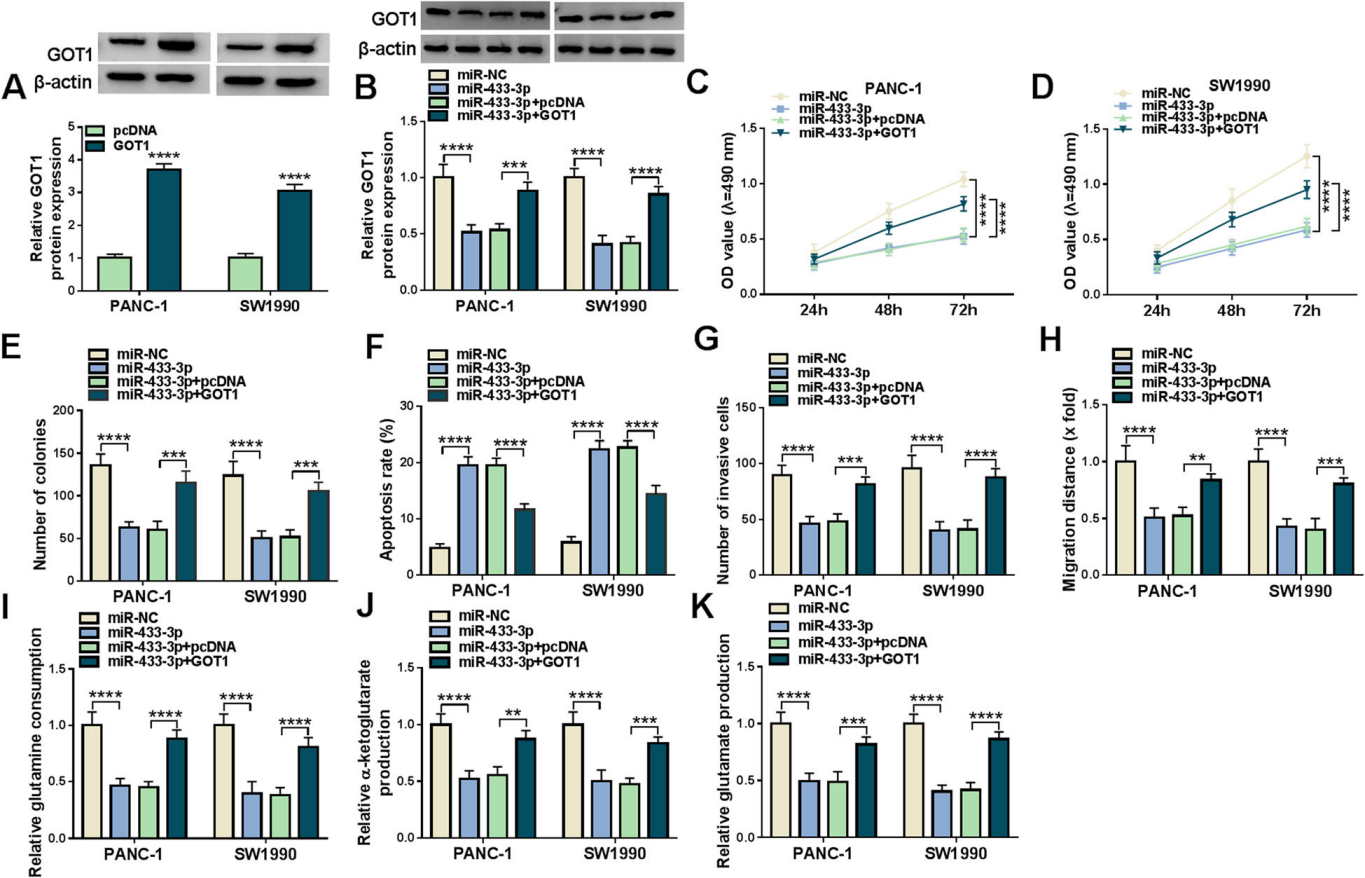
MiR-433-3p suppressed pancreatic cancer evolution via binding to GOT1. **a** The overexpression efficiency of GOT1 was determined by western blot in PANC-1 and SW1990 cells. **b** The impacts between miR-433-3p mimics and GOT1 overexpression on GOT1 protein expression were revealed by western blot analysis. **c**-**e** MTT and cell colony formation assays were employed to illustrate the effects between miR-433-3p mimics and GOT1 overexpression on cell proliferation in PANC-1 and SW1990 cells. **f** Flow cytometry analysis was used to determine the impacts between miR-433-3p mimics and enforced GOT1 expression on the apoptosis of PANC-1 and SW1990 cells. **g** and **h** Transwell invasion and wound-healing assays were performed to investigate the impacts between miR-433-3p mimics and GOT1 overexpression on the invasion and migration of PANC-1 and SW1990 cells. **i** and **j** Glutamine and α-KG assay kits were utilized to illustrate the influences between miR-433-3p and GOT1 on glutamine consumption and α-KG production, respectively, in PANC-1 and SW1990 cells. **k** Glutamate assay kit was used to explain the influences between miR-433-3p and GOT1 on glutamate production. The β-actin was employed for the normalization of GOT1. **P < 0.01, ***P < 0.001 and ****P < 0.0001

Given the binding relationship between miR-433-3p and GOT1, whether miR-433-3p regulated pancreatic cancer process by targeting GOT1 was continued to be explored. Results firstly presented GOT1 protein expression was dramatically upregulated in PANC-1 and SW1990 cells transfected with GOT1 as compared to the cells transfected with pcDNA (Fig. 7a), implicating the overexpression vector of GOT1 was successfully built. Subsequently, western blot showed that GOT1 overexpression restrained miR-433-3p mimic-mediated downregulation on GOT1 protein expression (Fig. 7b). MTT and cell colony formation assay displayed miR-433-3p repressed cell proliferation, whereas enforced GOT1 expression attenuated this impact (Fig. 7c-e). Additionally, miR-433-3p was found to induce cell apoptosis in PANC-1 and SW1990 cells, which was restored after GOT1 overexpression (Fig. 7f). The invasion and migration of PANC-1 and SW1990 cells were also inhibited by miR-433-3p mimics; however, GOT1 overexpression hindered these influences (Fig. 7g and h). Furthermore, the repressive impacts of miR-433-3p mimics on glutamine consumption, α-KG production and glutamate production were reversed by GOT1 overexpression (Fig. 7i-k).

Next, we studied the effects of GOT1 overexpression on GOT1 knockdown-mediated pancreatic cancer cell processes. As shown in Figure S5A, GOT1 abolished the repression effect of GOT1 depletion on GOT1 protein expression. The impacts of GOT1 silencing on cell viability, colony-forming ability and apoptosis were also reversed after GOT1 overexpression (Figure S5B-E). Additionally, si-GOT1-mediated impacts on cell metastasis, glutamine consumption, α-KG production and glutamate production were also hindered by enforced GOT1 expression (Figure S5F-J). Summarily, these findings demonstrated miR-433-3p regulated pancreatic cancer progression and glutamine metabolism by targeting GOT1.

### Circ-MBOAT2 silencing downregulated GOT1 expression by sponging miR-433-3p

**Fig. 8:**
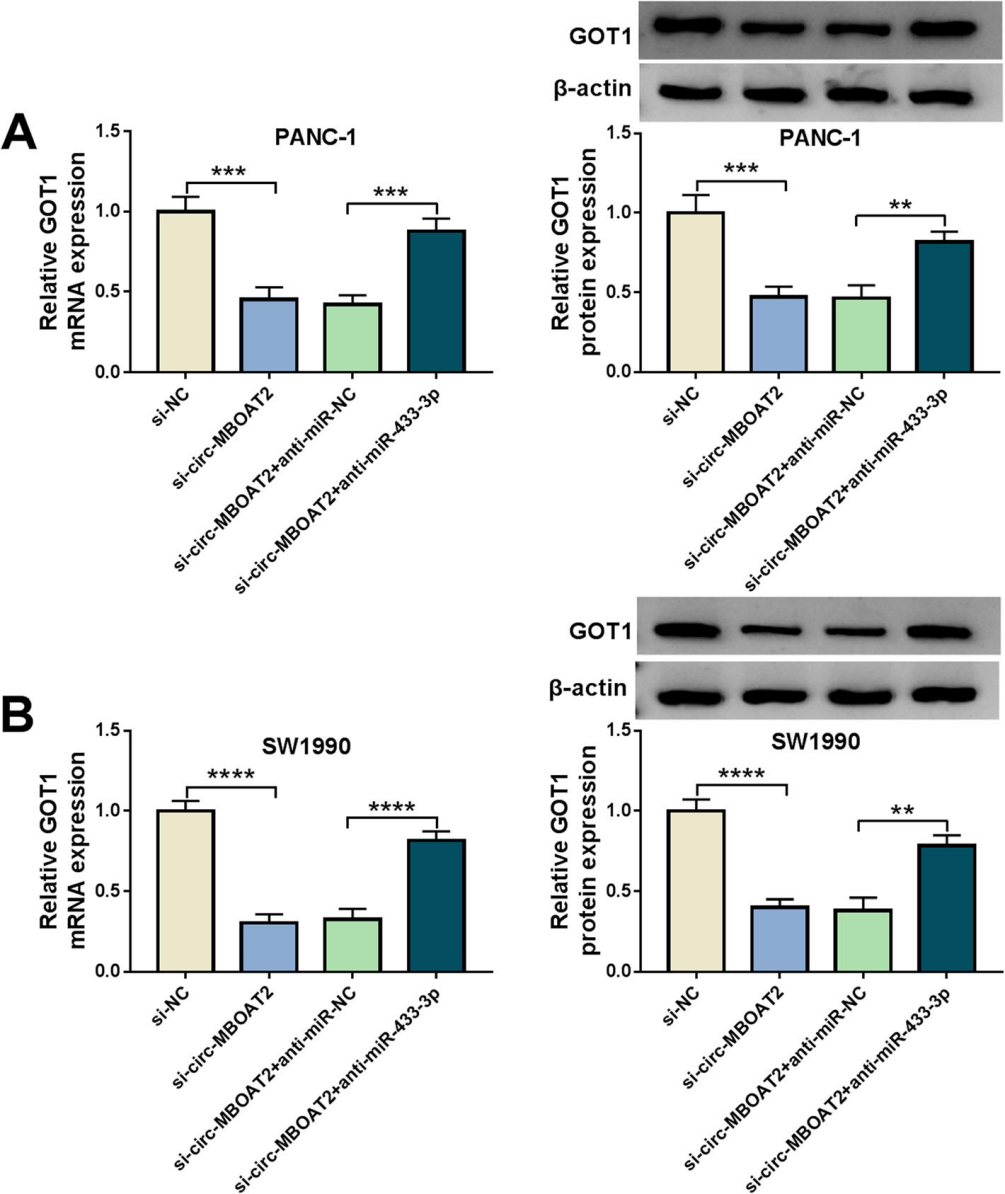
Circ-MBOAT2 regulated GOT1 expression by absorbing miR-433-3p. **a** and **b** The impacts between circ-MBOAT2 silencing and miR-433-3p inhibitors on the mRNA and protein expression of GOT1 were severally illustrated by qRT-PCR and western blot analysis in PANC-1 and SW1990 cells. The β-actin was employed for the normalization of GOT1. **P < 0.01, ***P < 0.001 and ****P < 0.0001

We have demonstrated circ-MBOAT2 was a sponge of miR-433-3p, which targeted GOT1. Thus, whether circ-MBOAT2 regulated GOT1 expression by sponging miR-433-3p was further revealed. Results exhibited the mRNA and protein levels of GOT1 were dramatically downregulated by circ-MBOAT2 silencing in PANC-1 and SW1990 cells; however, miR-433-3p inhibitors attenuated these effects (Fig. 8a and b). This result meant that circ-MBOAT2 could regulate GOT1 expression via sponging miR-433-3p.

## Discussion

CircRNAs, a novel RNA, are commonly dysregulated and play vital parts in pancreatic cancer development [24]. For example, circ-LDLRAD3 could serve as a diagnostic biomarker of pancreatic cancer as its close correlation with venous and lymphatic invasion as well as metastasis [25]. The immune escape of pancreatic cancer cells induced via hypoxia-inducible factor 1-alpha could be modulated by circ_0000977 [26]. In addition, circ_001653 downregulation hindered cell proliferative and invasive properties through sponging miR-377 [27]. In this research, we found circ-MBOAT2 silencing suppressed tumor progression and glutamine catabolism via downregulating GOT1 through sponging miR-433-3p.

Previous study has illustrated that circ-MBOAT2 acted as an oncogene in tumor growth in prostate cancer [28]. In addition, Yang et al. showed circ-MBOAT2 was highly expressed in pancreatic ductal cancer specimens, and it could increase metastasis–related matrix metallopeptidase 7 expression [29]. Herein, circ-MBOAT2 was also shown to be apparently upregulated in the tissue and cell samples of pancreatic cancer, and circ-MBOAT2 expression was higher in stage III-IV pancreatic cancer tissues than in stage I-II. It was found that circ-MBOAT2 silencing restrained cell proliferation, migratory and invasive abilities and glutamine catabolism, and circ-MBOAT2 overexpression abolished these effects. Additionally, circ-MBOAT2 knockdown upregulated cell apoptotic rate, and repressed tumor growth in vivo. The results from cytoplasmic and nuclear circ-MBOAT2 analysis displayed that circ-MBOAT2 was mainly located in cytoplasm, suggesting that circ-MBOAT2 chiefly functioned at the post-transcriptional level. Considering circRNAs serving as sponge of miRNAs to regulate cancer evolution [30], we further sought the miRNA associated with circ-MBOAT2.

Subsistent data unveiled that miR-433-3p participated in regulating the pathogenesis of various cancers with acting as an anti-oncogene. For instance, miR-433-3p hindered cell proliferative and metastatic capacities in esophageal squamous [31] and oral squamous cell cancer [32]. MiR-433-3p led to apparent repression on cell proliferation and metastasis, and promotion on cell apoptosis in lung cancer [33]. In this paper, qRT-PCR results showed that miR-433-3p expression was reduced in pancreatic cancer tissue samples and cell lines. In addition, miR-433-3p also be found to inhibit malignant properties of pancreatic cancer via repressing cell proliferation and metastasis as well as inducing cell apoptosis, which was consistent with the findings of Bi et al [20]. Beyond that, the role of miR-433-3p in glutamine catabolism was revealed in this paper. Data showed miR-433-3p repressed glutamine catabolism. Furthermore, miR-433-3p was negatively related to circ-MBOAT2 in expression, and miR-433-3p inhibitors attenuated si-circ-MBOAT2-mediated impacts on tumor development and glutamine catabolism. These evidences confirmed circ-MBOAT2 regulated malignant properties of pancreatic cancer by sponging miR-433-3p.

The downstream gene of miR-433-3p was continued to be explored. Results presented miR-433-3p contained the putative binding sites of GOT1, and the interaction between miR-433-3p and GOT1 was further identified by dual-luciferase reporter assay. Besides, our data displayed notably high GOT1 expression in PANC-1 and SW1990 cells. GOT1 ectopic expression restrained miR-433-3p-mediated influences on tumor development and glutamine catabolism, suggesting GOT1 could hinder malignant properties of pancreatic cancer, which was approved by the data from Wang et al [23]. Meanwhile, the above data demonstrated miR-433-3p modulated pancreatic cancer evolution via targeting GOT1.

The relationship between circ-MBOAT2 and GOT1 was further revealed. Data showed circ-MBOAT2 silencing decreased GOT1 expression, whereas miR-433-3p inhibitors impaired this impact. This finding implied circ-MBOAT2 could modulate GOT1 expression via sponging miR-433-3p.

All in all, we found circ-MBOAT2 silencing repressed pancreatic cancer progression via regulating cell proliferation, apoptosis, migration, invasion through repressing glutamine catabolism, and the underlying mechanism was that circ-MBOAT2 induced GOT1 expression via absorbing miR-433-3p (Figure S6). This finding not only provides a novel mechanism for studying circ-MBOAT2-based effects on the evolution of pancreatic cancer, but also provides a new potential biomarker for the diagnosis and therapy of pancreatic cancer.

## Conclusions

Interestingly, existing evidence has well documented the role of NRF2 in the regulation of glutamine catabolism that NRF2 can redirect glutamine into anabolic pathway [34]. Chio et al. indicated that NRF2 was necessary for the proliferation maintenance of pancreatic cancer cells [35]. Considering the importance of GOT1 in regulating glutamine catabolism, we hypothesized that GOT1-mediated pancreatic cancer progression was linked to NRF2. However, the detailed mechanism should be explored in subsequent study. Additionally, in view of relation of MBOAT with lysophospholipid acyltransferase [36], whether circ-MBOAT2 affected the acylation of lysophosphatidic acid into phosphatidic acid would be studied in future.

## Supplementary Information

**Additional file 1: Figure S1.** Circ-MBOAT2 expression was detected by qRT-PCR in stage I-II pancreatic cancer tissues (*N* = 22) and stage III-IV pancreatic cancer tissues (*N* = 12). TNM: tumor, node and metastasis. β-actin was employed for the normalization. ****P < 0.0001.

**Additional file 2: Figure S2.** The effect of circ-MBOAT2 silencing on MBOAT2 expression was determined by qRT-PCR in PANC-1 and SW1990 cells. Ns: no significance. The β-actin was employed for the normalization.

**Additional file 3: Figure S3.** Circ-MBOAT2 overexpression abolished si-circ-MBOAT2-mediated pancreatic cancer cell processes. (A-J) PANC-1 and SW1990 cells were transfected with si-NC, si-circ-MBOAT2, si-circ-MBOAT2 + pCD5-ciR and si-circ-MBOAT2 + circ-MBOAT2, respectively. (A) Circ-MBOAT2 expression was detected by qRT-PCR. (B-D) Cell viability and colony-forming ability were revealed by MTT and cell colony formation assays, respectively. (E) Flow cytometry analysis was used to determine cell apoptosis. (F and G) The invasion and migration of cells were demonstrated by transwell invasion and wound-healing assays, respectively. (H and I) Glutamine and α-KG assay kits were utilized to reveal glutamine consumption and α-KG production. (J) Glutamate assay kit was performed to detect glutamate production. **P < 0.01, ***P < 0.001 and ****P < 0.0001.

**Additional file 4: Figure S4.** The effects of circ-MBOAT2 knockdown on the expression of miR-144-3p and miR-433-3p were determined by qRT-PCR. The U6 was employed for the normalization. ***P < 0.001 and ****P < 0.0001.

**Additional file 5: Figure S5.** The effects between GOT2 overexpression and knockdown on pancreatic cancer cell processes. (A-J) PANC-1 and SW1990 cells were transfected with si-con, si-GOT1, si-GOT1 + pcDNA and si-GOT1 + GOT1, respectively. (A) GOT1 protein expression was quantified by western blot analysis. (B-D) Cell viability and colony-forming ability were revealed by MTT and cell colony formation assays, respectively. (E) Flow cytometry analysis was performed to quantify cell apoptosis. (F and G) The invasion and migration of cells were severally investigated by transwell and wound-healing assays. (Hand I) Glutamine and α-KG assay kits were utilized to reveal glutamine consumption and α-KG production. (J) Glutamate assay kit was performed to detect glutamate production. **P < 0.01, ***P < 0.001 and ****P < 0.0001.

**Additional file 6: Figure S6.** The schematic diagram of circ-MBOAT2-mediated pancreatic cancer progression.

## Data Availability

Not applicable.

## Abbreviations

circ-MBOAT2: circ-membrane bound O-acyltransferase domain containing 2
GOT1: Glutamic-oxaloacetic transaminase 1
α-KG: alpha ketoglutarate
