# Supplementary figures and images for: Circ-MBOAT2 knockdown represses tumor progression and glutamine catabolism by miR-433-3p/GOT1 axis in pancreatic cancer
Source: J Exp Clin Cancer Res. 2021 Apr 8;40:124. doi: 10.1186/s13046-021-01894-x (PMC8034179; doi:10.1186/s13046-021-01894-x)

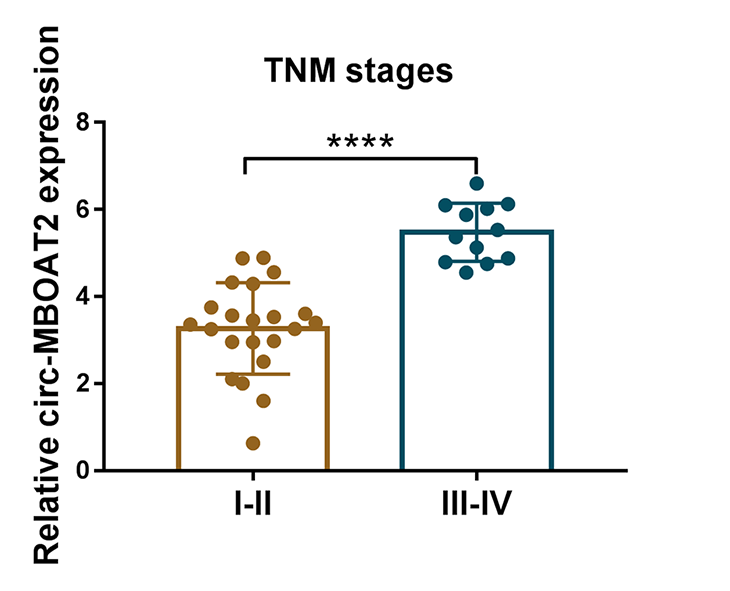

Supplement: Supplementary file 1 — Additional file 1: Figure S1. Circ-MBOAT2 expression was detected by qRT-PCR in stage I-II pancreatic cancer tissues (N = 22) and stage III-IV pancreatic cancer tissues (N = 12). TNM: tumor, node and metastasis. β-actin was employed for the normalization. ****P < 0.0001. [file 13046_2021_1894_MOESM1_ESM.tif]

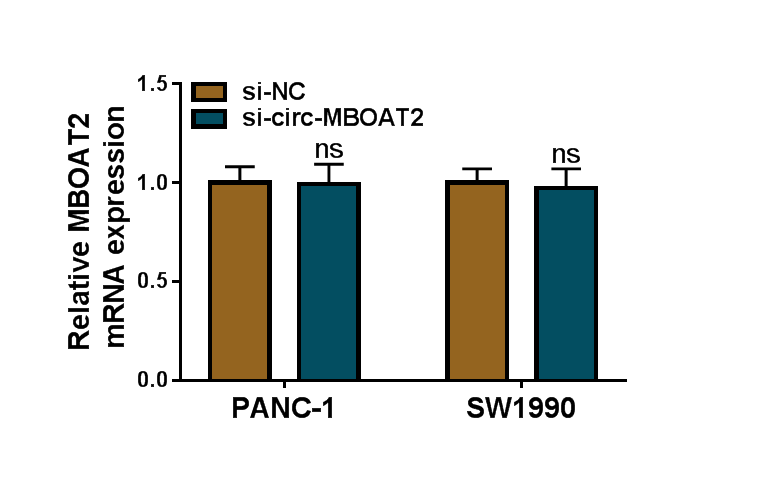

Supplement: Supplementary file 2 — Additional file 2: Figure S2. The effect of circ-MBOAT2 silencing on MBOAT2 expression was determined by qRT-PCR in PANC-1 and SW1990 cells. Ns: no significance. The β-actin was employed for the normalization. [file 13046_2021_1894_MOESM2_ESM.tif]

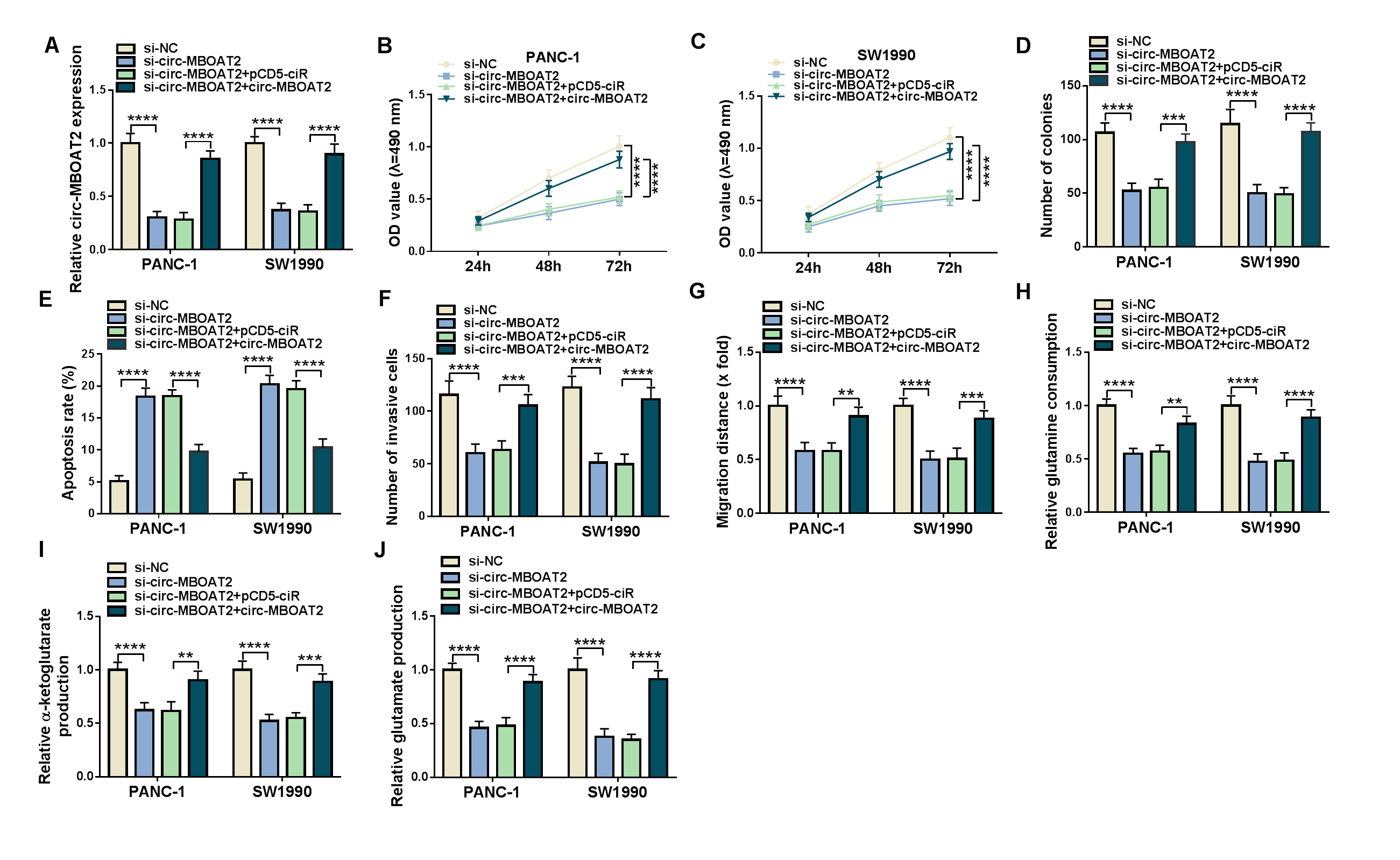

Supplement: Supplementary file 3 — Additional file 3: Figure S3. Circ-MBOAT2 overexpression abolished si-circ-MBOAT2-mediated pancreatic cancer cell processes. (A-J) PANC-1 and SW1990 cells were transfected with si-NC, si-circ-MBOAT2, si-circ-MBOAT2 + pCD5-ciR and si-circ-MBOAT2 + circ-MBOAT2, respectively. (A) Circ-MBOAT2 expression was detected by qRT-PCR. (B-D) Cell viability and colony-forming ability were revealed by MTT and cell colony formation assays, respectively. (E) Flow cytometry analysis was used to determine cell apoptosis. (F and G) The invasion and migration of cells were demonstrated by transwell invasion and wound-healing assays, respectively. (H and I) Glutamine and α-KG assay kits were utilized to reveal glutamine consumption and α-KG production. (J) Glutamate assay kit was performed to detect glutamate production. **P < 0.01, ***P < 0.001 and ****P < 0.0001. [file 13046_2021_1894_MOESM3_ESM.tif]

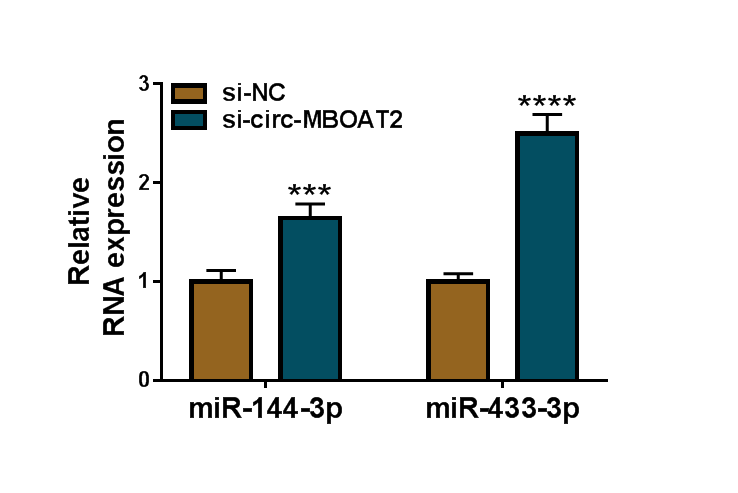

Supplement: Supplementary file 4 — Additional file 4: Figure S4. The effects of circ-MBOAT2 knockdown on the expression of miR-144-3p and miR-433-3p were determined by qRT-PCR. The U6 was employed for the normalization. ***P < 0.001 and ****P < 0.0001. [file 13046_2021_1894_MOESM4_ESM.tif]

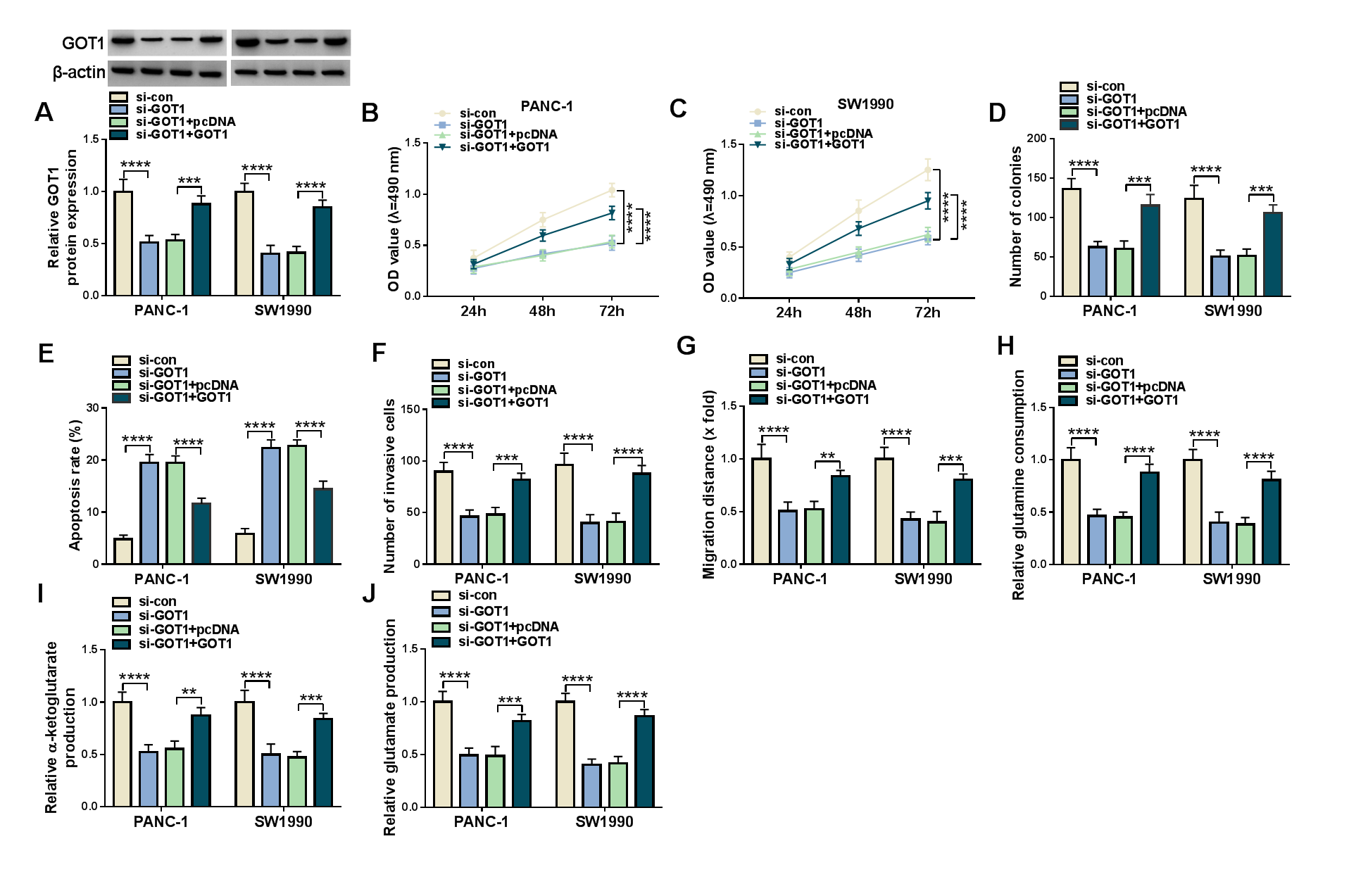

Supplement: Supplementary file 5 — Additional file 5: Figure S5. The effects between GOT2 overexpression and knockdown on pancreatic cancer cell processes. (A-J) PANC-1 and SW1990 cells were transfected with si-con, si-GOT1, si-GOT1 + pcDNA and si-GOT1 + GOT1, respectively. (A) GOT1 protein expression was quantified by western blot analysis. (B-D) Cell viability and colony-forming ability were revealed by MTT and cell colony formation assays, respectively. (E) Flow cytometry analysis was performed to quantify cell apoptosis. (F and G) The invasion and migration of cells were severally investigated by transwell and wound-healing assays. (Hand I) Glutamine and α-KG assay kits were utilized to reveal glutamine consumption and α-KG production. (J) Glutamate assay kit was performed to detect glutamate production. **P < 0.01, ***P < 0.001 and ****P < 0.0001. [file 13046_2021_1894_MOESM5_ESM.tif]

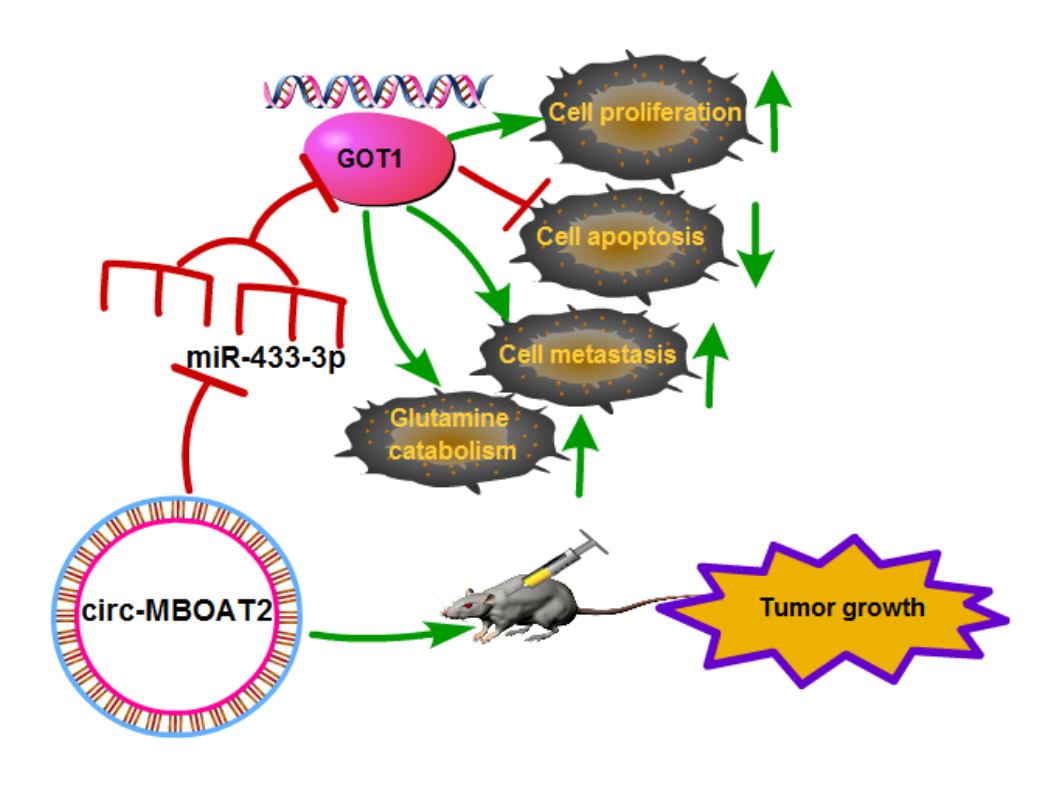

Supplement: Supplementary file 6 — Additional file 6: Figure S6. The schematic diagram of circ-MBOAT2-mediated pancreatic cancer progression. [file 13046_2021_1894_MOESM6_ESM.tif]
